# Effect of Postmenopausal Hormone Therapy on Metabolic Syndrome and Its Components

**DOI:** 10.3390/jcm13144043

**Published:** 2024-07-10

**Authors:** Young Hye Cho, Sang Yeoup Lee

**Affiliations:** 1 Department of Family Medicine and Biomedical Research Institute, Pusan National University Yangsan Hospital, Yangsan 50612, Republic of Korea; younghye82@naver.com; 2 Department of Family Medicine, Pusan National University School of Medicine, Yangsan 50612, Republic of Korea; 3 Department of Medical Education, Pusan National University School of Medicine, Yangsan 50612, Republic of Korea

Menopause is defined as the permanent cessation of ovarian function in women, typically occurring between the ages of 45 and 55. The loss of estrogen during menopause causes vasomotor symptoms, such as hot flashes and night sweats, as well as emotional changes including anxiety and depression. Additionally, it increases the long-term risk of osteoporosis and cardiovascular disease [1]. Menopause is also associated with an increase in body fat mass, particularly visceral fat, and a decline in metabolic function, leading to conditions such as insulin resistance and elevated cholesterol levels [1,2,3].

A meta-analysis comparing pre- and postmenopausal differences in the components of metabolic syndrome found that postmenopausal women had significantly more unfavorable changes in the components of metabolic syndrome, including waist circumference, low-density lipoprotein cholesterol (LDL-C), triglyceride (TG), blood pressure, and fasting glucose [3]. The study by Ou et al. in this Special Issue4 showed an increase in the prevalence of impaired glucose tolerance and hypertriglyceridemia after menopause without an increase in abdominal obesity or hypertension. These findings emphasize that while the changes in each component of metabolic syndrome may vary between studies, the overall prevalence of metabolic syndrome increases after menopause. In addition, active interventions are required to reduce the risk of cardiovascular disease in postmenopausal women. 

Menopausal hormone therapy (MHT) is indicated for treating postmenopausal symptoms, including vasomotor symptoms, and for preventing osteoporosis in women at high risk for osteoporotic fractures. Ou et al. [4] showed the possible association between MHT and a lower prevalence of metabolic syndrome in naturally menopausal women. This suggests that MHT may positively affect each component of metabolic syndrome.

Several studies have explored the relationship between MHT and components of metabolic syndrome, with mixed results regarding its effects on weight. In a previous cohort study including 1053 women aged 50 to 80, it was found that current users of MHT had the lowest body fat mass and the smallest increase in visceral fat mass at a 10-year follow-up, compared to past users and those who had never used MHT [5]. On the contrary, a Cochrane review concluded that hormone therapy did not affect body weight and body fat mass [6]. Despite these inconsistencies, MHT is generally associated with improved insulin resistance and a reduced risk of developing type 2 diabetes [7,8]. The protective mechanism of MHT against type 2 diabetes in postmenopausal women may involve maintaining insulin secretion and delaying diabetes onset by alleviating age-related estrogen receptor stress in pancreatic β-cells [9]. 

MHT showed favorable effects on the lipid profiles of postmenopausal women, except for increased TG levels. A recent meta-analysis found that MHT was associated with a significant reduction in total cholesterol, LDL-C, and lipoprotein (a) compared with placebo or no treatment [10]. These lipid profile improvements suggest that MHT could play a role in reducing cardiovascular risk in postmenopausal women.

Furthermore, women undergoing surgical menopause have a higher incidence of metabolic syndrome than those undergoing natural menopause, likely due to the earlier onset of menopause and rapid estrogen decline3. Ou et al. [4] substantiated that the effect of MHT on metabolic risk may differ in natural versus surgical menopause and may depend on the type and duration of MHT. These findings support personalized approaches to MHT administration tailored to the type of menopause.

While these findings are promising, further prospective studies are required to confirm the optimal MHT protocols for managing metabolic syndrome and reducing cardiovascular risk in postmenopausal women. The study by Ou et al. contributes to the growing body of evidence that MHT can be an effective strategy for improving metabolic health in postmenopausal women. Nevertheless, it also underscores the need for continued research to refine these interventions. 

In summary, menopause significantly impacts women’s metabolic health, increasing the risk of metabolic syndrome and cardiovascular disease. MHT offers a potential intervention to improve these risks, particularly its effects on body composition, insulin resistance, and lipid profiles. However, the variability in study outcomes and the differences in responses between natural and surgical menopause highlight the importance of individualized treatment plans and the need for ongoing research to optimize MHT use.
